# Child neurocognitive functioning influences the effectiveness of specific techniques in behavioral teacher training for ADHD: Moderator analyses from a randomized controlled microtrial

**DOI:** 10.1002/jcv2.12032

**Published:** 2021-10-16

**Authors:** Anouck I. Staff, Jaap Oosterlaan, Saskia van der Oord, Marsh Königs, Barbara J. van den Hoofdakker, Marjolein Luman

**Affiliations:** ^1^ Department of Clinical‐, Neuro‐, and Developmental Psychology Vrije Universiteit Amsterdam Amsterdam The Netherlands; ^2^ Emma Neuroscience Group Department of Pediatrics Amsterdam Reproduction & Development Emma Children's Hospital Amsterdam UMC University of Amsterdam Amsterdam The Netherlands; ^3^ Faculty of Psychology and Educational Sciences KU Leuven Leuven Belgium; ^4^ Department of Developmental Psychology University of Amsterdam Amsterdam The Netherlands; ^5^ Department of Child and Adolescent Psychiatry University Medical Center Groningen University of Groningen Groningen The Netherlands; ^6^ Department of Clinical Psychology and Experimental Psychopathology University of Groningen Groningen The Netherlands

**Keywords:** ADHD, antecedent‐based techniques, behavioral teacher training, consequent‐based techniques, microtrial, neurocognitive functioning

## Abstract

**Background:**

Childhood attention‐deficit/hyperactivity disorder (ADHD) is associated with several neurocognitive impairments. Whether these impairments influence the effectiveness of techniques that are commonly used in behavioral teacher training for ADHD has not been investigated so far.

**Method:**

In this microtrial, teachers of 90 children with ADHD symptoms (6–12 years) were randomly assigned to a short intervention consisting of either antecedent‐based (stimulus‐control) techniques or consequent‐based (contingency management) techniques, or to a waitlist control condition. Primary outcome was the daily assessment of individually selected problem behavior, assessed pre‐ and post‐intervention. Potential neurocognitive moderators of treatment effect included teacher ratings of cognitive control, reward, and punishment sensitivity, and measures derived from computerized neurocognitive tasks, including attentional lapses, interference control, visuospatial working memory, and emotional functioning. Intervention condition by moderator interactions were assessed in separate multilevel mixed models.

**Results:**

Lapses of attention, working memory, and emotional functioning interacted with intervention effectiveness. Antecedent‐based techniques were effective independent of these neurocognitive functions; consequent‐based techniques were (more) effective when these functions were more impaired. The effectiveness of techniques was neither related to interference control nor to teacher‐rated neurocognitive functioning.

**Conclusions:**

This study showed that child neurocognitive functioning influences the effectiveness of behavioral teacher techniques for children with ADHD symptoms. Findings suggest that antecedent‐based techniques may be effective for all children, while consequent‐based techniques have added value particularly for children who suffer from low visuospatial working memory, low emotional functioning, and/or large numbers of attentional lapses.

## Graphical abstract

Behavioral teacher training is an effective intervention to improve behavior of children with ADHD symptoms at school, but more insight in subgroups who are more or less susceptible to these treatments is needed. This study investigated the influence of child neurocognitive functioning on the effectiveness of antecedent‐ (i.e., stimulus‐control) and consequent‐based (i.e., contingency management) techniques in behavioral teacher training for ADHD. Antecedent‐based techniques were effective irrespective of child neurocognitive functioning, whilst consequent‐based techniques were particularly effective for children with relatively large numbers of lapses of attention, lower visuospatial working memory or lower emotional functioning abilities.

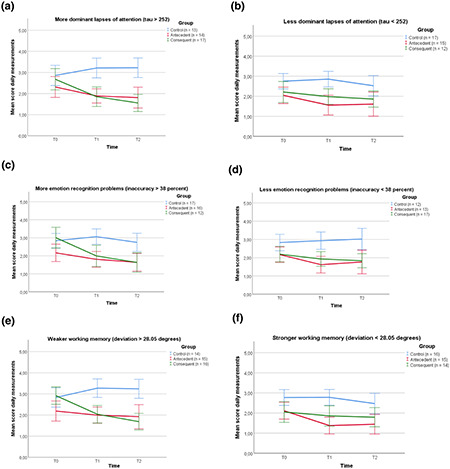

**Key points**

- Antecedent‐based (i.e., stimulus‐control) techniques in behavioral teacher training for attention‐deficit/hyperactivity disorder (ADHD) were effective in improving classroom problem behavior irrespective of child neurocognitive functioning.
- Consequent‐based (i.e., contingency management) techniques were particularly effective for children with relatively large numbers of attentional lapses, lower visuospatial working memory, or lower emotional functioning abilities.
- Findings suggest that interventions may always include antecedent‐based techniques, while consequent‐based techniques may have added value particularly for children who suffer from low neurocognitive functioning.

## INTRODUCTION

Attention‐deficit/hyperactivity disorder (ADHD) is a common childhood mental disorder, with approximately 5% of children meeting full criteria for the disorder (Polanczyk et al., 2014), and 10%–15% experiencing impairing levels of symptoms without meeting full diagnostic criteria (Kirova et al., 2019). Both groups of children suffer from impairments at home and in school, with academic impairment and social problems being most prominent in the school situation.

Behavioral teacher training is an effective non‐pharmacological intervention to improve behavior of children with ADHD at school. Within such interventions, teachers are taught different techniques, commonly including both antecedent‐based (i.e., stimulus‐control) and consequent‐based (i.e., contingency management) techniques. Antecedent‐based techniques aim at establishing and strengthening the association between antecedents and the desired behavior of the child (Van der Oord & Tripp, 2020) by manipulating the antecedents of a child's behavior. Consequent‐based techniques are aimed at strengthening the association between the child's behavior and its positive or negative consequences (Van der Oord & Tripp, 2020) by manipulating the consequences of a child's behavior. Nevertheless, effect sizes of behavioral teacher interventions are medium at best and effectiveness differs substantially between individuals (Evans et al., 2018), suggesting that there may be subgroups of children that benefit more or less from such interventions. Recent emphasis on tailoring interventions to individual needs (i.e., personalized treatment) has led research to shift its attention from interventions targeted at specific categories (i.e., a disorder‐based approach) to adapting interventions to cognitive processes that may underlie mental disorders, such as neurocognitive functioning (Cuthbert, 2014; Faraone et al., 2015).

There is little work on the impact of child neurocognitive functioning on behavioral intervention effectiveness for ADHD so far. For the current study, we were interested in looking at whether core neurocognitive problems reported for children with ADHD impact on the effectiveness of intervention components. First, children with ADHD often show impairments in working memory, in particular visuospatial working memory (Martinussen et al., 2005), and interference control (Willcutt et al., 2005), both needed for cognitive control. Second, temporal information processing problems have been observed in ADHD, comprising difficulties in temporal representations (i.e., time estimation and production), which contribute to intra‐individual variability in responses and manifest in dominant lapses of attention (Toplak et al., 2006). Third, altered motivational processes (i.e., reward and punishment sensitivity) have been observed ADHD: shown by an enlarged dependency on external rewards in order to shape behavior (Luman et al., 2010). Fourth, and more recently, emotional functioning, including impairments in recognizing emotional expressions of others, has been identified as impaired neurocognitive function in ADHD (Sjöwall et al., 2013). There is large heterogeneity in the neurocognitive impairments seen in children with ADHD, with most children showing impairments in one or two neurocognitive functions, but there is also a subgroup of children without impairments (Faraone et al., 2015). In children with subthreshold ADHD, similar but milder, neurocognitive impairments have been observed (Salum et al., 2014).

Although the role of child neurocognitive functioning in the effectiveness of behavioral teacher techniques has not been investigated thus far, one might argue that antecedent‐based techniques, such as providing clear structure and behavioral expectations, while decreasing distracting stimuli, unburden neurocognitive functions such as cognitive control and/or timing difficulties and prevent problem behavior from occurring (Nigg, 2017). One might also suggest that discussing difficult situations ahead can prevent inappropriate behavior in children experiencing impairments in emotional functioning. For consequent‐based techniques, one might hypothesize that altered reinforcement learning could be scaffolded by providing consequences in a frequent, immediate, and consequent manner (Van der Oord & Tripp, 2020). As a result, it may be that children with impairments in cognitive control, temporal processing, and emotional functioning would benefit from antecedent‐based techniques, while children with alterations in reinforcement learning would profit from consequent‐based techniques. However, experimental studies in this area have not been done so far.

The current study aimed to investigate whether child neurocognitive functioning moderated the effectiveness of behavioral teacher training techniques. A recent study by our group has shown that antecedent‐ and consequent‐based techniques were equally and highly effective in reducing ADHD behaviors in the classroom when compared to a waitlist control condition (Staff, Van Den Hoofdakker, et al., 2021). In the current study, we conducted a head‐to‐head comparison of the antecedent‐ and consequent‐based techniques and explored the possible moderating role of visuospatial working memory, interference control, reward and punishment sensitivity, lapses of attention and emotional functioning abilities. Neurocognitive functions were assessed by computerized neurocognitive tasks and teacher rating scales, since both measures may reflect different aspects of neurocognitive functioning (Soto et al., 2020; Toplak et al., 2013).

## METHOD

### Design

For a detailed description of design and randomization of the study, please see Staff, Van Den Hoofdakker, et al. (2021). Teachers were trained in either antecedent‐ or consequent‐based techniques, or were allocated to a waitlist control condition (i.e., 30 children and their teachers per condition). To investigate the moderating role of child neurocognitive functioning on technique effectiveness, a randomized controlled microtrial was used. Microtrials are randomized experiments testing the immediate effects of relatively brief and focused environmental manipulations on proximal outcomes (Howe et al., 2010). Such a design is specifically useful, given its power to detect moderation effects (Howe & Ridenour, 2019). Outcome measures were assessed at baseline prior to randomization (T0), during the week immediately after the two intervention sessions, or the 2‐week waiting period (T1), and 3 weeks after the intervention or waiting period (T2). This study was registered at the Dutch Trial Register: https://www.trialregister.nl/trial/6616 (trial registration number: NL6616).

### Participants

Teachers of 90 children with teacher‐rated ADHD symptoms and aged between 6 and 12 years old (regular primary education, grade 1–6) from schools in rural and urban areas in the Netherlands, participated in this study. Participating teachers were seeking help to cope with the behavior of one or two of their students showing ADHD symptoms. For the inclusion criteria, see Staff, Van Den Hoofdakker, et al. (2021). In short, children were included if they (a) obtained a score >90th percentile on one of the ADHD scales of the teacher version of the Disruptive Behavior Disorders Rating Scale (DBDRS) (Oosterlaan et al., 2008), (b) showed at least three symptoms on one of the ADHD scales of the DSM‐IV‐TR based Teacher Telephone Interview (TTI) (Tannock et al., 2002), and (c) showed impairment on at least one domain of the teacher Impairment Rating Scale (IRS) (Fabiano et al., 2006). Children were excluded if they (a) had a full scale IQ < 70, estimated using a short version of the Wechsler Intelligence Scale for Children—third edition (WISC‐III‐NL), (b) were taking psychotropic medication during the last month, (c) had a diagnosis of autism spectrum disorder or conduct disorder as reported by parents, or (d) if the teacher had received a behavioral training in the past year. The CONSORT flowchart presented in Figure S1 displays the inclusion process of participants.

### Interventions

We investigated two short, protocolized interventions (two individual teacher sessions), focusing on either antecedent‐ or consequent‐based techniques. In short, the antecedent intervention focused on how stimuli evoke behavior, how cognitive control deficits and lack of sense of time in children with ADHD may lead to difficulties in adapting their behavior to stimuli, and how teachers may use antecedent‐based techniques to alter stimuli and influence behavior prior to its onset. In the consequent intervention, teachers were taught how consequences affect behavior, how altered reinforcement learning may influence the way the child's behavior is shaped by the environment, and how consequent‐based techniques may be used following (un)desired behavior to affect the occurrence of behavior. One or more techniques of the particular set were chosen to be part of the intervention plan, based on a behavioral analysis for targeted problem behaviors (see below). Intervention fidelity was good. For more information on the interventions fidelity please see Appendix S1.

### Outcome measures

#### Daily measures of problem behavior

Primary outcome was the daily assessment of four preselected individual problem behaviors related to ADHD in a specific situation (i.e., inattentive, hyperactive, impulsive, and oppositional behaviors), selected from a list of 32 behaviors. Internal consistency of this list in the current sample was excellent (*α* = 0.90) (Staff, Van Den Hoofdakker, et al., 2021). Two behaviors were targeted in the intervention and generalization may occur to the other two behaviors. Teachers rated daily, for each of the four behaviors, whether the behavior occurred that day (*yes*, or *no* = 0) and if yes, they scored the severity of the behavior on a 5‐point Likert scale (range 1–5). The mean score over four problem behaviors on five consecutive days served as dependent variable and were assessed pre‐ (T0) and post‐intervention (T1, T2). Please see Staff, Van Den Hoofdakker, et al. (2021) for detailed information on the assessment procedure.

#### Neurocognitive tasks

##### Lapses of attention and interference control

A modified version of the Flanker Task (Eriksen & Eriksen, 1974) was used to assess lapses of attention (tau) and interference control. Tau was calculated from the exponential component of the reaction time (RT) distribution fitted on the 48 neutral trials (‐‐>‐‐) of the task (Massidda & Massidda, 2013), with higher values of tau reflecting a greater number of attentional lapses. Interference control was calculated by the difference between inverse efficiency scores (mean RT/proportion correct) calculated on 48 incongruent (<<><<) and 48 congruent (>>>>>) trials (Mullane et al., 2009), with higher scores indicating poorer interference control.

##### Visuospatial working memory

An adaptation of the paradigm designed by Burnett Heyes et al. (2012) was used to assess visuospatial working memory precision. Here, 60 memory recall trials were used. Dependent measure was the deviation between the target stimulus (one of two randomly oriented colored bars) and the response stimulus (a bar with the same color as one of the target stimuli), which had to be rotated to match the orientation of the target stimulus (expressed in degrees). Higher scores reflected less accurate working memory precision.

##### Emotional functioning

Emotional functioning, specifically emotion recognition, was assessed by the Morphed Facial Emotion Recognition Task (MFERT) (Staff, Luman, et al., 2021). Children had to indicate the corresponding emotion condition when presented with 126 pictures of child faces displaying emotional expressions (happy, sad, angry, fearful) in different intensities (20%–100%), or neutral expressions. Dependent measure was the percentage of incorrect responses across emotion conditions and intensity levels, with higher scores reflecting less accurate emotion recognition.

#### Teacher ratings of neurocognitive performance

##### Cognitive control

The Cognition (13 items) and the Self‐direction and Organization (17 items) subscales of the Cognition and Motivation in Everyday Life (CAMEL) rating scale (Van Liefferinge et al., 2017) were used to assess cognitive control. Outcome was the mean score across both scales (range 0–4), with higher scores indicating more difficulties with cognitive control.

##### Reinforcement sensitivity

Reinforcement sensitivity was assessed using the Reward Responsivity (7 items) and Punishment Sensitivity (15 items) scales of the Dutch Sensitivity to Punishment and Sensitivity to Reward Questionnaire for Children (SPSRQ‐C) (Luman et al., 2012). Teachers rated child behavior on a 5‐point Likert scale (range 1–5). The mean score served as outcome, with higher scores indicating a heightened sensitivity to rewards or punishment.

### Procedure

The study was conducted between April 2017 and April 2019. Teachers were informed about the research aims and responsibilities of all parties involved. Subsequently, teachers informed parents about the study. Written consent was obtained from teachers, parents, and children older than 11 years. Baseline assessments took place during 1 week (T0). A research assistant visited the school to administer the neurocognitive test battery to the child. After baseline assessments were completed, children were randomized to one of the three intervention conditions. The local medical ethical committee waived the need for medical ethical approval (University Medical Center Groningen, 2016/198).

### Statistical analysis

Data were analyzed on an intention‐to‐treat basis. Outliers (>3SD) were winsorized (Tabachnick et al., 2007). Groups randomized to the three conditions were compared on neurocognitive outcomes by independent samples *t*‐tests. To test whether neurocognitive functioning influences the effectiveness of the two sets of techniques, multilevel analyses (mixed model) comparing both intervention conditions were conducted in Stata. Intervention condition (antecedent‐, consequent‐based) was inserted as between‐subjects factor, and time (T1, T2) as within‐subject variable. Baseline scores (T0) were inserted as fixed factor, in order to control for possible differences in daily rated problem behaviors at baseline. Interactions between the intervention effect (averaged over T1 and T2) and the potential moderator (i.e., neurocognitive functions) were added to the multilevel model one by one. Benjamini‐Hochberg correction was applied for multiple testing (i.e., seven moderators) (Thissen et al., 2002). Although our previous work has demonstrated that antecedent‐ and consequent‐based techniques are both highly effective when compared to a waitlist control condition (Staff, Van Den Hoofdakker, et al., 2021), we checked whether, in case of a moderating effect, the intervention was (still) more effective than waitlist control for the different levels of the moderator (based on median‐split analysis). Given that the development of neurocognitive functioning is age dependent (Diamond, 2013), age was inserted as fixed factor in the model in all analyses. Effect sizes (Cohen's *d*) were calculated by dividing the difference in mean scores between two conditions averaged over T1 and T2 by the pooled SD, with 0.20, 0.50, and 0.80 as thresholds for small, medium, and large effects.

## RESULTS

Demographic characteristics of the sample are presented in Table S2. Participants in the three intervention conditions did not differ on any of the inclusion characteristics (*p* > .121), with the exception of hyperactivity/impulsivity symptoms that were lower in participants in the antecedent condition compared to those in the consequent (TTI) and waitlist control condition (TTI and DBDRS). Parents reported that 23 children (26%) had been clinically diagnosed with ADHD. Based on the TTI, 42 children (47%) met the criteria for a diagnosis of ADHD. None of the parents indicated that children had received a diagnosis of oppositional defiant disorder (ODD), but 10 children (11%) met ODD criteria as indicated on the TTI.

### Moderators of technique effectiveness

**FIGURE 1:**
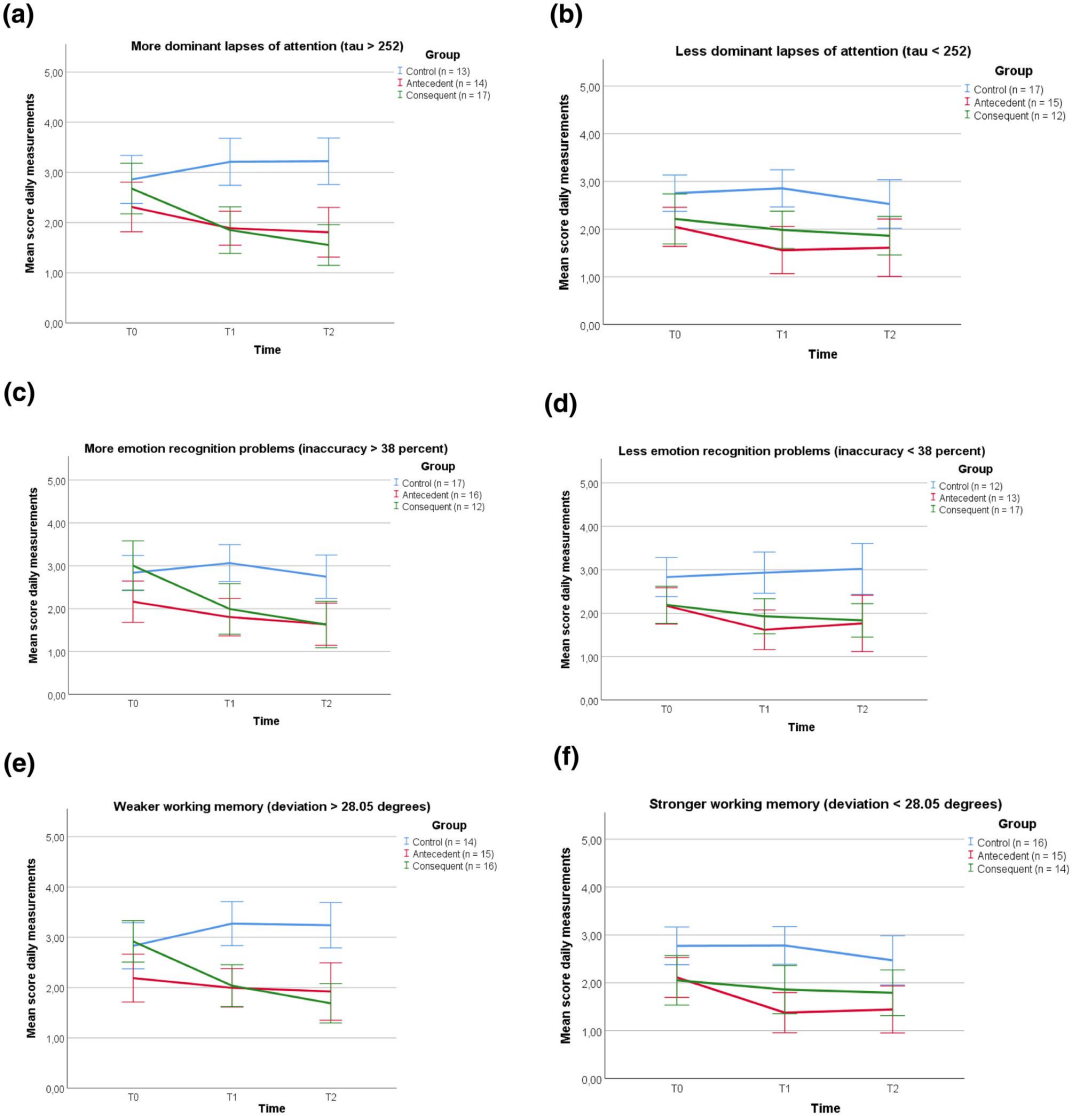
Observed values for the development of problem behavior over time in the three conditions, split on low and high levels of the moderators lapses of attention (panels a and b), emotional functioning (panels c and d), and working memory (panels e and f). The left panels represent lower performances on neurocognitive functioning tasks, the right panels represent higher performances. Daily measurement scores are means across four behaviors measured on five consecutive days. Error bars represent 95% confidence intervals

Results of the multilevel analyses are depicted in Table 1 and Figure 1. No interactions between teacher‐rated neurocognitive measures and intervention conditions were observed when comparing antecedent‐ and consequent‐based techniques, indicating that the effectiveness of both sets of techniques was independent of impairment in cognitive control, reward and punishment sensitivity as rated by teachers. For neurocognitive task performance, results revealed that lapses of attention, emotional functioning, and visuospatial working memory interacted with intervention condition on the reduction of problem behavior from pre‐ to post‐intervention. The effectiveness of techniques was not related to interference control.

**TABLE 1:** Results of the separate multilevel moderator analyses on daily rated problem behaviors

	Teacher ratings of neurocognitive performance						Neurocognitive tasks							
	Cognitive control^a^		Reward sensitivity^a^		Punishment sensitivity^a^		Lapses of attention^b^		Interference control^b^		Working memory^a^		Emotional functioning^c^	
	*B*(SE)	*p*	*B*(SE)	*p*	*B*(SE)	*p*	*B*(SE)	*p*	*B*(SE)	*p*	*B*(SE)	*p*	*B*(SE)	*p*
Constant	0.37 (0.62)	.555	0.43 (0.67)	.517	0.18 (0.59)	.756	1.54 (0.64)	.017	0.69 (0.50)	.166	0.64 (0.66)	.332	−2.68 (0.73)	<.001
Baseline (T0)	0.53 (0.08)	<.001	0.52 (0.08)	<.001	0.50 (0.08)	<.001	0.55 (0.08)	<.001	0.50 (0.08)	<.001	0.58 (0.07)	<.001	0.60 0(.08)	<.001
Age	−0.01 (0.04)	.843	0.02 (0.04)	.698	−0.02 (0.04)	.594	−0.01 (0.04)	.754	−0.01 (0.04)	.820	0.05 (0.05)	.289	−0.04 (0.04)	.295
Moderator	0.08 (0.12)	.514	−0.02 (0.15)	.915	0.29 (0.21)	.167	<0.01 (<0.01)	.004	<0.01 (0.01)	.599	−0.03 (0.01)	.024	−0.05 (0.01)	.001
Group	0.50 (0.54)	.361	−0.40 (0.75)	.598	0.01 (0.62)	.992	−1.22 (0.44)	.006	−0.04 (0.17)	.829	−1.53 (0.42)	<.001	−1.52 (0.71)	.032
Group x Moderator	−0.16 (0.17)	.368	0.13 (0.21)	.546	−0.05 (0.26)	.847	0.01 (<0.01)^d^	.002	0.01 (0.01)	.656	0.06 (0.01)^d^	<.001	0.04 (0.02)^d^	.015

*Note*: Coefficients and SE are depicted. The fixed effect of group represent group differences (antecedent vs. consequent) averaged over T1 and T2 while controlling for baseline scores (T0). The consequent condition was used as reference group.

^a^ *N* = 60.

^b^ *N* = 59: one child (antecedent condition) did not successfully pass practice trials.

^c^ *N* = 58: data of two children (one in each condition) are missing.

^d^ Significant after Benjamini‐Hochberg correction.

Post‐hoc analyses for lapses of attention and emotional functioning revealed that in the antecedent condition, the decrease in problem behavior during the intervention period was unrelated to neurocognitive task performance (*B* < 0.001, SE < 0.001, *p* = .712; *B* = −0.01, SE = 0.01, *p* = .337, respectively). In the consequent condition, however, the decrease in problem behavior during the intervention period was larger for children who showed more lapses of attention (*B* < −0.001, SE < 0.001, *p* < .001) and/or more problems with emotional functioning (*B* = −0.04, SE = 0.01, *p* < .001). The median‐split analyses with lapses of attention revealed that, for children with fewer lapses of attention (tau < 252), the consequent condition did not significantly differ from the control condition (*B* = −0.34, SE = 0.18, *p* = .065, *d* = 0.35), while for children with more lapses of attention (tau > 252), the consequent condition was still effective (*B* = 1.52, SE = 0.22, *p < *.001, *d* = 1.48). For emotional functioning problems, irrespective of emotional functioning ability, the consequent intervention was effective compared to the control condition, although the effect size was large for children with lower emotional functioning abilities and medium‐sized for children with higher abilities (inaccuracy>38; *B* = 1.18, SE = 0.26, *p* < .001, *d* = 1.14; inaccuracy<38; *B* = 0.67, SE = 0.20, *p* = .001, *d* = 0.68).

For working memory, children with higher working memory (deviation < 28.05) revealed larger intervention effects in the antecedent than consequent condition (*B* = −0.44, SE = 0.19, *p* = .019, *d* = 0.45). For children with lower working memory (deviation > 28.05), the effect was in the opposite direction: the consequent intervention was more effective than the antecedent intervention (*B* = 0.50, SE = 0.23, *p* = .027, *d* = 0.51) (all effects controlled for baseline differences in problem behaviors, see *Statistical analyses*). For children with higher working memory, the consequent intervention did not significantly differ from the control condition (*B* = −0.32, SE = 0.19, *p* = .104, *d* = 0.32), while for children with lower working memory, the antecedent intervention remained significantly effective (*B* = −0.98, SE = 0.23, *p* < .001, *d* = 0.98).

Figure 1 illustrates the results of the median‐split analyses, showing that within the consequent condition the decrease of problem behavior is larger when neurocognitive functions are lower. This figure also indicates that in the control condition, children with lower working memory and/or lapses of attention showed a deterioration in problem behavior over time (*B* = 0.02, SE = 0.01, *p* = .016; *B* < 0.001, SE < 0.001, *p* = .024, respectively).

To get more insight into associations between these three neurocognitive outcomes as well as with other relevant variables, correlations between the primary outcome, symptom severity and all neurocognitive outcomes were calculated. Table S3 displays these results, showing that correlations between the three moderators were medium‐sized (*r* = −0.41–0.68) and that these impairments were not related to ADHD and ODD symptom severity (*r* < 0.18). A principal component analysis (PCA) based on the correlation matrix revealed that all three neurocognitive outcomes loaded high on the same component (0.72–0.88), together explaining 68% of the variance.

## DISCUSSION

This study showed that effectiveness of antecedent‐ and consequent‐based techniques were moderated by child impairments in computer‐based lapses of attention, visuospatial working memory, and emotional functioning. Whereas antecedent‐based techniques were effective independent of these neurocognitive functions, consequent‐based techniques were (more) effective when these functions were more impaired. The effectiveness of both sets of techniques were unrelated to child impairments in computer‐based interference control and/or teacher‐rated neurocognitive functioning.

Although earlier attempts to identify child neurocognitive functioning as moderators on the effectiveness of behavioral parent training revealed mixed results (e.g., Adalio et al., 2018; van Langen et al., 2020), our results show the importance of neurocognitive functioning in the effectiveness of behavioral teacher training techniques. Our study differs from previous work in that we differentiated between the sets of techniques within behavioral training for ADHD, and found that, while all children benefited from antecedent‐based techniques, children with lower neurocognitive functions benefited more, or even only, from consequent‐based techniques. Children with these impairments also deteriorated over time in the control condition. This finding that problem behavior of children with more severe problems, in this case low neurocognitive functioning, worsened over time, stresses the importance of immediate intervention delivery for this group to protect them against increase of problems while waiting for treatment (Groenman et al., 2021).

Further, given that children without specific neurocognitive impairments did not appear to benefit from consequent‐based techniques, it may be that antecedent‐based techniques are suitable to use for all children, while consequent‐based techniques seem of particular value for children with lower neurocognitive functions. Our findings suggest that children with neurocognitive impairments in working memory, emotional functioning, and attentional lapses seem more dependent on external reinforcement such as salient consequences (e.g., compliments from the teacher) to learn what behavior is expected, and they may also need more salient consequences to learn the association between a stimulus and desired behavior (Luman et al., 2010). Children without these problems may learn stimulus–response associations without additional reinforcement provided by the teacher. For example, for emotional functioning, this may indicate that children experiencing difficulties in recognizing emotions need both structure regarding what behavior is expected and salient consequences to their behavior in order to show or maintain adequate (social) behavior. Nevertheless, further research including a combined antecedent‐ and consequent‐based intervention (in line with what is currently often provided in clinical practice) is needed to conclude whether the efficacy of such combined intervention is influenced by neurocognitive performance of the child. This may also include follow‐up assessments to gain insight into the role of child neurocognitive functioning on longer term intervention effects.

Given the medium‐sized inter‐correlations between lapses of attention, working memory, and emotional functioning, one may speculate about an overlapping etiological (brain) mechanism that may further explain the moderating effects of neurocognitive functioning in the effectiveness of consequent‐based techniques. The PCA showed that there may be an underlying neurocognitive factor that may explain our findings regarding these three inter‐correlated neurocognitive functions. We propose two hypotheses regarding this factor. First, given that children with lower neurocognitive functions respond in a similar fashion to the sets of techniques as compared to younger children (i.e., consequent‐based techniques are particularly effective; Staff, Van Den Hoofdakker, et al., 2021), current findings may indicate that children with lower neurocognitive functions experience a neurocognitive maturational lag (Shaw et al., 2007). Second, observed weaknesses on these three neurocognitive functions may occur as a result of failures to effectively switch between resting and active cognitive state, which may compete with goal‐directed activities and the performance of complex tasks (Diamond, 2013; Sonuga‐Barke & Castellanos, 2007).

We assessed neurocognitive functioning by rating scales and performance‐based measures. Our findings show that the latter seem more sensitive and/or ecologically valid for assessing susceptibility to intervention techniques in children with ADHD symptoms. This may be explained by attentional lapses, working memory, and emotional functioning not being easily observed by the teacher or are salient in a classroom situation. Nevertheless, such an extensive test battery may be less suitable for clinical practice.

Despite the promising directions for future research, there are limitations to consider. First, we had a relatively small sample. Although microtrials are suitable to detect moderation effects in small samples (Howe & Ridenour, 2019), weaker effects may have remained undetected due to limited power. Further, given the relatively small sample, we only assessed one neurocognitive function at a time and were not powered to identify whether there are neurocognitive profiles of children for whom techniques are more or less effective. Second, we only included a teacher‐rated measure of reinforcement learning, while results on performance‐based measures suggest that the latter are more sensitive to detect moderation effects. Results should be replicated in larger samples with multiple measures assessing several neurocognitive functions to draw more strong conclusions on the role of neurocognitive functioning in the effectiveness of techniques. Third, our sample mainly consisted of children with subthreshold ADHD and low levels of ODD symptoms. Since children with ADHD and comorbid ODD show different neurocognitive deficits compared to ADHD‐only children (Noordermeer et al., 2020), these results may not be generalized to samples with more severe ADHD and/or comorbid ODD. Fourth, given that we have investigated sets of techniques, we cannot conclude on which specific technique of a particular set is most likely to have affected the decrease in problem behavior. For example, whether planned ignorance of unwanted behavior or praise of desired behavior has caused the decrease of problem behavior remains unclear. Fifth, some of the measures employed lack a normative base wherefore we could not identify whether results were indeed below average in the study group compared to the general population. Taken together, this study is a first attempt to assess the impact of child neurocognitive functioning into technique effectiveness in behavioral teacher training. Our findings suggest that antecedent‐based techniques may be effective independent of neurocognitive functioning of the child, while consequent‐based techniques may have added value particularly in children with lower neurocognitive functions. Further research in larger (and possibly more severe) samples is needed to draw more strong conclusions on the role of neurocognitive functioning of children in the impact of different behavioral techniques on their behavior. Future studies should also examine whether interventions tailored to neurocognitive impairments are more effective than traditional, “one‐size‐fits‐all” interventions in reducing ADHD behavior and whether moderation effects are obtained on broader ADHD outcomes (e.g., DSM‐based questionnaires). The current results provides first insights into directions to personalize teacher interventions based on neurocognitive impairments of the child.

## CONFLICT OF INTEREST

The authors report no conflict of interest.

## ETHICS STATEMENT

Written consent was obtained from teachers, parents, and children older than 11 years.

## AUTHOR CONTRIBUTIONS

Anouck I. Staff: Conceptualization; Data curation; Formal analysis; Investigation; Methodology; Project administration; Resources; Software; Supervision; Validation; Visualization; Writing—original draft. Jaap Oosterlaan: Formal analysis; Funding acquisition; Methodology; Resources; Software; Supervision; Validation; Writing—review and editing. Saskia van der Oord: Conceptualization; Funding acquisition; Methodology; Resources; Software; Supervision; Validation; Writing—review and editing. Marsh Königs: Formal analysis; Methodology; Resources; Software; Supervision; Validation; Writing—review and editing. Barbara van den Hoofdakker: Conceptualization; Funding acquisition; Methodology; Resources; Supervision; Validation; Writing—review and editing. Marjolein Luman: Conceptualization; Formal analysis, Funding acquisition; Investigation; Methodology; Resources; Supervision; Validation; Writing—review and editing.

## Supporting information

Supporting Information S1

TABLE S1

TABLE S2

TABLE S3

FIGURE S1

FIGURE S2

## Data Availability

The data that support the findings of this study are available from the corresponding author (AS), upon reasonable request.
