# Supplementary material for: Child neurocognitive functioning influences the effectiveness of specific techniques in behavioral teacher training for ADHD: Moderator analyses from a randomized controlled microtrial
Source: JCPP Adv. 2021 Oct 16;1(3):e12032. doi: 10.1002/jcv2.12032 (PMC10242932; doi:10.1002/jcv2.12032)
Supplement: Supplementary file 1 — Supporting Information S1 [file JCV2-1-e12032-s003.docx]

**Supporting Information File S1.** Detailed information on the teacher interventions and intervention fidelity.

This supporting information is taken from Staff et al. (2021).

**Methods**

*Interventions*

Two short interventions were developed: an antecedent-based intervention using only antecedent-based techniques and a consequent-based intervention using only consequent-based techniques. The interventions consisted of two individual sessions with the teacher by a trained psychologist in two consecutive weeks. The first session lasted two hours and took place at school, and the second session lasted 45 minutes, and took place by video conference. After each session, teachers were instructed to implement the techniques in the classroom for four weeks, and teachers could consult the therapist if required. These protocollized interventions were based on evidence-based behavioral parent training programs (Barkley, 1987; McMahon & Forehand, 2003; Van Den Hoofdakker et al., 2007) aimed at remediating ADHD and ADHD related behaviors, and consisted of psycho-education on ADHD, selecting and analyzing specific problem behavior, and training of stimulus control and contingency management techniques.

Common elements in both interventions

Interventions were targeted at two out of four preselected problem behaviors from a list of 32 ADHD-related behaviors (see below), with both sessions targeting one selected problem behavior in one specifically chosen situation (see below) (e.g., difficulties staying focused during individual seatwork). The other two problem behaviors were not directly targeted in the intervention. The first session started with psycho-education about ADHD behaviors. Thereafter, one problem behavior was selected by the teacher in collaboration with the therapist, based on the severity and frequency of occurrence (preferably daily) of the problem behavior. Depending on the intervention condition (see below), the therapist and teacher made a behavioral analysis by identifying (a) antecedents that elicit the problem behavior, or (b) consequences that positively or negatively reinforce the problem behavior (i.e., functional behavior assessment, FBA; Dunlap & Kern, 2018). In the next step they defined desired target behavior. The therapist and teacher made a behavioral intervention plan with antecedent- or consequent-based techniques, depending on the assigned intervention condition. This procedure allowed us to have an individually tailored intervention plan for both problem behaviors of every child. The session ended with practicing the technique(s) through role-play and/or visualization. The teacher implemented the plan in the classroom for one week, after which the second session took place. This session started with evaluating the preceding week and adapting the intervention plan, if necessary. Thereafter, the therapist and teacher selected a second problem behavior occurring in a specific situation, and went through the same steps as in the first session (i.e., from behavioral analysis to practicing). The teacher received handouts containing a summary of the specific techniques trained.

Antecedent-based intervention

In the antecedent-based intervention (referred to as antecedent condition), teachers were taught how stimuli evoke behaviors and how executive and timing (i.e. planning) deficits in children with ADHD may lead to difficulties adapting behavior to stimuli. The therapist and teacher identified which antecedents elicited the selected problem behavior. Thereafter, teachers were taught how antecedent-based techniques may be used prior to the onset of behavior and how to alter stimuli in order to elicit changes in child behaviors (Owens et al., 2018). Given the experimental set-up of the microtrial that was aimed to study specific intervention components, teachers were only taught antecedent-based techniques in the antecedent condition. The following techniques were briefly explained in this intervention: setting clear rules, providing clear instructions, discussing challenging situations with the child in advance, and providing structure in time and space. One or more techniques were chosen to be part of the intervention plan, based on the behavioral analysis. When teachers brought up that they could use techniques from the other condition (e.g., reward desired behavior), the therapists were instructed to respond that that is a known technique, but that the current training focused on implementing antecedent-based techniques first.

As an example, a desired target behavior may be: ‘This child can work individually for five minutes on the assigned math task, without asking the teacher for help’. In the antecedent condition, the intervention plan may consist of (1) the teacher giving appropriate individual instructions to the child after the class wide instruction; (2) the child having a step-by-step plan with illustrations (i.e., pictograms) on how to proceed the task and what to do when a question arises; and (3) a timer on the child’s desk to show the remaining time.

Consequent-based intervention

In the consequent-based intervention (referred to as consequent condition), teachers were taught how consequences affect behavior and that children with ADHD may suffer from an altered reward sensitivity that may influence how their behavior is shaped by the environment. The therapist and teacher identified which consequences positively or negatively reinforced or discouraged desired target behavior. Thereafter, teachers were taught how consequent-based techniques may be used following (un)desired behavior to affect the occurrence of specific behavior. The following techniques were briefly explained in this intervention: praise, reward, planned ignoring and negative consequences. When the full desired behavior was not yet displayed by the child, shaping was explained (rewarding of short sequences of the desired behavior) with the aim to elicit the full desired behavior. Consequent-based techniques such as token economy and time-out were not taught in this intervention given that these techniques also require antecedent-based techniques (e.g., clear rules, discussing in advance). As in the antecedent-based intervention, techniques for the intervention plan were chosen based on problem behavior and behavioral analysis, no antecedent-based techniques were taught, and when teachers suggest to add an antecedent-based technique to the intervention plan they were told to focus on consequent-based techniques first.

As an example, a desired target behavior may be: ‘This child can work individually for five minutes on the assigned math task, without asking the teacher for help’. The intervention plan in the consequent condition may consist of (1) the teacher frequently rewarding the child’s (attempts to display the) desired behavior (e.g., praise or thumbs up when the child is working or quiet); (2) the teacher praising other children who are working on their task; and 3) the teacher ignoring all of the child’s attention-seeking behavior (e.g., raising hand, calling the teacher’s name).

Therapists

Two psychologists with postgraduate training in behavioral therapy and ADHD and trained in using the intervention protocol (AS and RH), carried out the intervention. To assess intervention fidelity, we measured therapists’ adherence to the protocol based on contamination and the percentage addressed session items. Contamination was assessed using the procedures of Abikoff (Abikoff et al., 2013), and defined as therapists’ actions that resulted in the incorporation of features from the non-assigned intervention (e.g., consequent) into the assigned intervention (e.g., antecedent). This could occur either by the therapist recommending the use of non-assigned techniques or the therapist actively supporting and elaborating on the teacher’s suggestions of the use of techniques specific to the non-assigned intervention. The frequency of contamination occurrences in a session served as the outcome. We also assessed the percentage of addressed session items in each session (18 items in session one, 11 items in session two). Furthermore, all intervention sessions were audiotaped. For every therapist, the first session of both conditions were checked on intervention fidelity by two of the authors who are behavior therapists and licensed supervisors in the postgraduate behavior therapy program with ample experience in behavioral parent and teacher training programs (SvdO and BvdH). Fidelity scores were discussed with the therapist during individual supervision sessions. Further, ten percent of the sessions were listened back and scored on intervention fidelity during the study by independent evaluators. After each session, the therapists completed a fidelity checklist in which they were asked which items were covered. During the entire study, therapists and researchers held meetings every two weeks to monitor intervention fidelity and to provide supervision.

**Results on intervention fidelity**

*Intervention fidelity*

Therapist reported fidelity

Therapists’ reports of fidelity showed that on average respectively 98.9% and 99.4% (*SD* = 2.26, *N* = 29; *SD* = 1.37, *N* = 30) of the session items were carried out in the sessions of the antecedent and consequent condition. Fidelity did not differ between the two intervention conditions (*t*(45.77) = -1.13, *p* = .266).

Recorded fidelity

Based on the recorded sessions, average protocol adherence was high in both conditions (antecedent = 98.0%, consequent = 97.8%). Contamination occurred once in a session of the consequent-based intervention and was not scored in any of the antecedent-based sessions. Contamination did not differ between the two interventions (*t*(3.00) = -1.00, *p* = .391).

**References**

Abikoff, H. B., Gallagher, R., Wells, K. C., Murray, D. W., Huang, L., Lu, F., & Petkova, E. (2013). Remediating organizational functioning in children with ADHD: Immediate and long-term effects from a randomized controlled trial. *Journal of consulting and clinical psychology, 81*(1), 113.

Barkley, R. A. (1987). *Defiant children*: Guilford press New York, NY, USA:.

Dunlap, G., & Kern, L. (2018). Perspectives on Functional (Behavioral) Assessment. *Behavioral Disorders, 43*(2), 316-321.

McMahon, R., & Forehand, R. (2003). Helping the noncompliant child: A clinician’s guide to effective parent training. *New York: Guilford*.

Owens, J. S., Holdaway, A. S., Smith, J., Evans, S. W., Himawan, L. K., Coles, E. K., . . . Dawson, A. E. (2018). Rates of common classroom behavior management strategies and their associations with challenging student behavior in elementary school. *Journal of Emotional and Behavioral Disorders, 26*(3), 156-169.

Staff, A. I., Van Den Hoofdakker, B. J., Van der Oord, S., Hornstra, R., Hoekstra, P. J., Twisk, J. W. R., . . . Luman, M. (2021). Effectiveness of specific techniques in behavioral teacher training for childhood ADHD: A randomized controlled microtrial. *Journal of Clinical Child & Adolescent Psychology*. doi:10.1080/15374416.2020.1846542

Van Den Hoofdakker, B. J., Van der Veen-Mulders, L., Sytema, S., Emmelkamp, P. M., Minderaa, R. B., & Nauta, M. H. (2007). Effectiveness of behavioral parent training for children with ADHD in routine clinical practice: a randomized controlled study. *Journal of the American Academy of Child & Adolescent Psychiatry, 46*(10), 1263-1271.
