# Supplementary material for: Child neurocognitive functioning influences the effectiveness of specific techniques in behavioral teacher training for ADHD: Moderator analyses from a randomized controlled microtrial
Source: JCPP Adv. 2021 Oct 16;1(3):e12032. doi: 10.1002/jcv2.12032 (PMC10242932; doi:10.1002/jcv2.12032)
Supplement: Supplementary file 2 — TABLE S1 [file JCV2-1-e12032-s001.docx]

**Supporting Information Table S1.**

| **Table S1**. Overview of the assessed rating scales and neurocognitive tasks. | | | |
| --- | --- | --- | --- |
| Measure and key reference | Neurocognitive function | Task description | Dependent measure |
| Performance-based tasks |  |  |  |
| Flanker task (Eriksen & Eriksen, 1974) | Lapses of attention | Children were presented with a target arrow centrally presented on a computer screen, flanked by arrows pointing in the same direction (congruent trials, e.g., >>>>>), in the opposite direction (incongruent trials, e.g., <<><<) or by horizontal rectangles (neutral trials, e.g., -->--). Children had to press one of two buttons, corresponding to the direction of the target arrow. They were instructed to respond as quickly and accurately as possible. The experimental task consisted of four blocks of 36 trials (i.e., 48 neutral, 48 congruent and 48 incongruent trials presented in a random order), with short breaks between each block. Total task duration was approximately nine minutes. The task has adequate reliability and validity (Fan, McCandliss, Sommer, Raz, & Posner, 2002). | Tau reflecting the exponential component of the RT distribution derived from 48 neutral trials (Massidda & Massidda, 2013). |
|  | Interference control |  | Difference in inverse efficiency score (mean RT divided by proportion correct) between 48 incongruent and 48 congruent trials (Mullane, Corkum, Klein, & McLaughlin, 2009). |
| VSWMP (Burnett Heyes, Zokaei, van der Staaij, Bays, & Husain, 2012) | Visuospatial working memory | Participants were presented with a fixation cross (1000ms), followed by two colored bars (stimuli, presented and then followed by a blank screen both 500ms). After this, a response bar was presented in the color matching of one of the stimulus bars. Children were instructed to manipulate the orientation of the response bar by moving the mouse along the y-axis, to match the orientation of the stimulus bar with the identical color. The task consists of 60 trials, presented in three blocks of 20 trials. In between blocks there is a fixed break of 60 seconds. Total task duration was 14 minutes. As working memory precision seems to be a more sensitive index of working memory abilities than the traditionally used number of items that can be stored (i.e., working memory capacity or span) (Zokaei, Burnett Heyes, Gorgoraptis, Budhdeo, & Husain, 2015). | Mean deviation in degrees between the target bar and response bar. |
| MFERT (Staff et al., 2021) | Emotional functioning | Following a fixation cross in the middle of the screen (250ms), participants were shown (400ms) a picture of a child face (three males, three females) displaying an emotional expression (happy, sad, angry, fearful) in intensity levels ranging between 20% and 100%, or a neutral expression. Neutral expressions of every child actor were morphed with high-intensity expressions for each emotion, resulting in 21 pictures per child actor: a neutral expression, and four emotional conditions expressed in five levels of expression intensity (e.g., an intensity level of 20% for the expression of anger consisted of a morphed picture with 80% neutral and 20% expression of anger, whereas the 100% intensity expression of anger consisted of 0% neutral and 100% anger). Children had to indicate the corresponding emotion condition by clicking on one of the five emotion labels (four emotions, one neutral) with a computer mouse. Response options remained visible on the screen throughout the task. The task consisted of 126 pictures randomly presented in two blocks of 63 trials, with a short break in between. The task lasted approximately 12 minutes. This is an ecologically valid task to measure emotion recognition that uses child faces instead of commonly used adult faces, and it discriminates between the four main emotions and multiple expression intensities. The task is able to discriminate between children with and without (subthreshold) ADHD and emotion recognition accuracy is related to social and emotional problems children with (subthreshold) ADHD experience (Staff et al., 2021). | Inaccuracy reflected in percentage of incorrect responses across emotion conditions and intensity levels. |
| Teacher ratings of neurocognitive performance |  |  |  |
| SPSRQ-C (Luman, van Meel, Oosterlaan, & Geurts, 2012) | Reward sensitivity | Reward Sensitivity was assessed using the Reward Responsivity scale of the SPSRQ-C. Teachers rated behavior (7 items) on a 5-point Likert scale (1 = ‘*strongly disagree*’, 5 = ‘*strongly agree*’). The parent-rated Reward Responsivity scale is reliable (*α* = .83), and differentiates between children with ADHD and typically developing peers, the teacher version has not been tested yet on reliability and validity (Luman et al., 2012). | Mean scale score. |
|  | Punishment sensitivity | Punishment Sensitivity was assessed using the Punishment Sensitivity scale of the SPSRQ-C. The scale consisted of 15 items and was rated by teachers. Scaling was similar to the Reward Sensitivity scale described above. The parent-rated Punishment Sensitivity scale is reliable (*α* = .86) and able to discriminate between children with ADHD and typically developing peers (Luman et al., 2012). | Mean scale score. |
| CAMEL (Van Liefferinge et al., 2017) | Cognitive control | Cognitive control was assessed using the Cognition and Self-direction and Organization scales of the CAMEL. The Cognition scale (13 items) measures aspects of working memory and planning. The Self-direction and Organization scale (17 items) assesses cognitive control functions as inhibition, organization and error-monitoring. Teachers rated behavior on a 5-point Likert scale ranging from 0 (‘*a lot less than average*’) to 4 (‘*a lot more than average*’). The psychometric properties of the parent version of the CAMEL are adequate, the teacher version has not been tested yet on reliability and validity (Van Liefferinge et al., 2017). | Mean score across both scales. |
| *Note*. CAMEL = Cognition And Motivation in Everyday Life rating scale; MFERT = Morphed Facial Emotion Recognition Task; RT = reaction time; SPSRQ-C = Sensitivity to Punishment and Sensitivity to Reward Questionnaire for Children; VSWMP = Visuospatial Working Memory Precision task. | | | |

**References**

Burnett Heyes, S., Zokaei, N., van der Staaij, I., Bays, P. M., & Husain, M. (2012). Development of visual working memory precision in childhood. *Developmental science, 15*(4), 528-539.

Eriksen, B. A., & Eriksen, C. W. (1974). Effects of noise letters upon the identification of a target letter in a nonsearch task. *Perception & psychophysics, 16*(1), 143-149.

Fan, J., McCandliss, B. D., Sommer, T., Raz, A., & Posner, M. I. (2002). Testing the efficiency and independence of attentional networks. *Journal of cognitive neuroscience, 14*(3), 340-347.

Luman, M., van Meel, C. S., Oosterlaan, J., & Geurts, H. M. (2012). Reward and punishment sensitivity in children with ADHD: validating the sensitivity to punishment and sensitivity to reward questionnaire for children (SPSRQ-C). *Journal of Abnormal Child Psychology, 40*(1), 145-157.

Massidda, D., & Massidda, M. D. (2013). Retimes: Reaction time analysis. R package version 0.1-2. Retrieved from <https://CRAN.R-project.org/package=retimes>.

Mullane, J. C., Corkum, P. V., Klein, R. M., & McLaughlin, E. (2009). Interference control in children with and without ADHD: a systematic review of Flanker and Simon task performance. *Child Neuropsychology, 15*(4), 321-342.

Staff, A. I., Luman, M., van der Oord, S., Bergwerff, C. E., van den Hoofdakker, B. J., & Oosterlaan, J. (2021). Facial emotion recognition impairment predicts social and emotional problems in children with (subthreshold) ADHD. *European child & adolescent psychiatry*, 1-13.

Van Liefferinge, D., Sonuga-Barke, E., Van Broeck, N., Van Der Oord, S., Lemiere, J., & Danckaerts, M. (2017). A rating measure of ADHD-related neuropsychological impairment in children and adolescents: Data from the Cognition and Motivation in Everyday Life (CAMEL) Scale from population and clinical samples. *Child Neuropsychology, 23*(4), 483-501.

Zokaei, N., Burnett Heyes, S., Gorgoraptis, N., Budhdeo, S., & Husain, M. (2015). Working memory recall precision is a more sensitive index than span. *Journal of neuropsychology, 9*(2), 319-329.
