# Supplementary material for: Child neurocognitive functioning influences the effectiveness of specific techniques in behavioral teacher training for ADHD: Moderator analyses from a randomized controlled microtrial
Source: JCPP Adv. 2021 Oct 16;1(3):e12032. doi: 10.1002/jcv2.12032 (PMC10242932; doi:10.1002/jcv2.12032)
Supplement: Supplementary file 3 — TABLE S2 [file JCV2-1-e12032-s004.docx]

**Supporting Information Table S2.**

| **Table S2.** Background characteristics of the sample. | | | | |
| --- | --- | --- | --- | --- |
|  | Antecedent  (*n*=30) | Consequent  (*n*=30) | Waitlist control  (*n*=30) | Group comparisons |
| Teachers (*n*) | 25 | 26 | 26 |  |
| Schools (*n*) | 17 | 18 | 17 |  |
| *Child factors* |  |  |  |  |
| Age in years | 8.53 (1.63) | 9.08 (1.63) | 8.76 (1.52) | *F*(2,89)=.88, *p*=.420 |
| Sex, *n* (*%*) boys | 23 (77) | 23 (77) | 28 (93) | *χ*^2^=3.81, *p*=.150 |
| IQ | 99.77 (11.04) | 99.33 (14.28) | 104.07 (10.05) | *F*(2, 89) = 1.45, *p* = .241 |
| SES*^a^* | 5.22 (1.24) | 5.24 (1.12) | 5.00 (1.03) | *F*(2, 88) = .41, *p* = .664 |
| Caucasian, *n* (*%*) | 28 (93) | 27 (90) | 30 (100) | *Fisher’s exact* = .294, *p =* .363 |
| ADHD diagnosis, *n* (*%*) | 8 (27) | 8 (27) | 7 (23) | *χ*^2^ = .12, *p* = .943 |
| Other psychiatric diagnosis, *n* (*%*) | 0 (0) | 3 (10)*^b^* | 0 (0) | *Fisher’s exact =* 4.22, *p =* .104 |
| TTI symptom severity  Inattention  Hyperactivity/Impulsivity    ODD  CD | 4.30 (1.58)  2.97 (1.85)  1.10 (1.45)  .00 (.00) | 5.00 (1.86)   - 1. (2.38)   2. (1.46)   .00 (.00) | 4.13 (1.91)  4.60 (2.22)  1.23 (1.61)  .13 (.51) | *F*(2, 89) = 1.99, *p* = .143  *F*(2, 89) = 6.65, *p* = .002  (CC, WC > AC)  *F*(2, 89) = .10, *p* = .903  *F*(2, 89) = 2.07, *p* = .132 |
| DBDRS  Inattention  Hyperactivity/Impulsivity | 16.90 (4.96)  13.17 (6.21) | 17.50 (3.92)  15.73 (6.49) | 16.00 (5.57)  17.57 (6.60) | *F*(2, 89) = .72, *p* = .488  *F*(2, 89) = 3.54, *p* = .033 (WC > AC) |
| IRS impairment  Number of domains  Average score | 3.07 (.98)  6.22 (1.65) | 2.97 (1.27)  6.14 (1.97) | 3.24 (.88)  6.29 (1.28) | *F*(2, 84) = .45, *p* = .638  *F*(2, 84) = .52, *p* = .948 |
| Daily ratings of problem behavior at T0 | 2.15 (.79) | 2.51 (.93) | 2.80 (.74) | *F*(2, 89) = 4.63, *p* = .012 |
| *Teacher ratings of neurocognitive performance* |  |  |  |  |
| Cognitive control (CAMEL, range 0-4) | 3.08 (.89) | 2.84 (.99) | 3.01 (.85) | *F*(2,89)=.55, *p*=.580 |
| Reward sensitivity (SPSRQ-C, range 1-5) | 3.52 (.79) | 3.58 (.75) | 3.64 (.76) | *F*(2,89)=.20, *p*=.821 |
| Punishment sensitivity (SPSRQ-C, range 1-5) | 2.58 (.69) | 2.09 (.54) | 2.12 (.49) | *F*(2,89)=6.74, *p*=.002 (AC > CC, WC) |
| *Neurocognitive tasks* |  |  |  |  |
| Lapses of attention (tau on Flanker Task) | 252.76 (103.22)*^c^* | 253.20 (85.51) | 238.97 (118.97) | *F*(2,89)=.18, *p*=.833 |
| Interference control (∆ RT/% correct on Flanker Task) | 5.78( 11.74)*^c^* | 1.25 (16.95) | -.31 (14.27) | *F*(2,89)=1.40, *p*=.251 |
| Working memory (∆ degrees on VSWMP) | 30.58 (10.86) | 27.31 (9.77) | 26.92 (10.80) | *F*(2,89)=1.09, *p*=.340 |
| Emotional functioning (% incorrect on MFERT) | 42.94 (10.58)*^d^* | 37.95 (8.07)*^d^* | 39.92 (9.93)*^d^* | *F*(2,89)=1.99, *p*=.143 |
| *M* and *SD* are depicted unless otherwise stated*.*  *Note*. AC = antecedent condition; ADHD = attention-deficit/hyperactivity disorder; CAMEL=Cognition And Motivation in Everyday Life rating scale; CC = consequent condition; CD = conduct disorder; DBDRS = Disruptive Behavior Disorder Rating Scale; MFERT=Morphed Facial Emotion Recognition Task; ODD = oppositional defiant disorder; RT=reaction time; SES = socioeconomic status; SPSRQ-C=Sensitivity to Punishment and Sensitivity to Reward Questionnaire for Children; TTI = Teacher Telephone Interview; VSWMP=Visuospatial Working Memory Precision task; WC = waitlist control condition.  *^a^* SES was measured by parental educational level (average of both parents) through the Dutch classification system (1 = no education completed, 2 = early childhood education, 3 = primary education, 4 = lower secondary education, 5 = upper secondary education, 6 = undergraduate school, 7 = graduate school, 8 = post-graduate education) (CBS, 2016).  *^b^* Learning disorder  *^c^*One child did not successfully pass practice trials; *n*=29.  *^d^*Data of one child is missing; *n*=29. | | | | |

**References**

CBS. (2016). *Standaard onderwijsindeling 2016 (The Dutch Standard Classification of Education)*. Den Haag: Dutch Central Bureau of Statistics.
