# Supplementary material for: Child neurocognitive functioning influences the effectiveness of specific techniques in behavioral teacher training for ADHD: Moderator analyses from a randomized controlled microtrial
Source: JCPP Adv. 2021 Oct 16;1(3):e12032. doi: 10.1002/jcv2.12032 (PMC10242932; doi:10.1002/jcv2.12032)
Supplement: Supplementary file 4 — TABLE S3 [file JCV2-1-e12032-s006.docx]

**Supporting Information Table S3.**

| **Table S3**. Correlation matrix of the primary outcome, symptom severity and neurocognitive functioning outcomes. | | | | | | | | | |  |
| --- | --- | --- | --- | --- | --- | --- | --- | --- | --- | --- |
|  | 1. | 2. | 3. | 4. | 5. | 6. | 7. | 8. | 9. | 10. |
| 1. Daily ratings of problem behaviors at T0 | - | .266* | .163 | -.277** | .058 | -.034 | .132 | -.080 | .126 | .142 |
| 2. ADHD symptom severity (TTI) |  | - | .081 | -.253* | .089 | -.143 | .177 | -.043 | .091 | .101 |
| 3. ODD symptom severity (TTI) |  |  | - | .153 | .239* | -.272** | -.084 | .006 | -.073 | -.143 |
| 4. Cognitive control (CAMEL) |  |  |  | - | .235* | -.147 | -.234* | .023 | -.319** | -.240* |
| 5. Reward sensitivity (SPSRQ-C) |  |  |  |  | - | -.188 | -.249* | .098 | -.186 | -.174 |
| 6. Punishment sensitivity (SPSRQ-C) |  |  |  |  |  | - | .058 | .040 | .120 | .123 |
| 7. Lapses of Attention (Flanker)*^a^* |  |  |  |  |  |  | - | -.063 | .667** | .412** |
| 8. Interference Control (Flanker)*^a^* |  |  |  |  |  |  |  | - | .081 | -.095 |
| 9. Working memory (VSWMP) |  |  |  |  |  |  |  |  | - | .460** |
| 10. Emotional functioning (MFERT) |  |  |  |  |  |  |  |  |  | - |
| *Note*. *N* = 90. *^a^N* = 89,  *^b^N* = 88. Pearson correlations are depicted. **p* < .05, ***p* < .01. ADHD = attention-deficit/hyperactivity disorder; CAMEL = Cognition and Motivation in Everyday Life rating scale; MFERT = Morphed Facial Emotion Recognition Task; ODD = oppositional defiant disorder; SPSRQ-C = Sensitivity to Punishment and Sensitivity to Reward Questionnaire for Children; TTI = Teacher Telephone Interview; VSWMP = Visuospatial Working Memory Precision task. | | | | | | | | | |  |
