# Supplementary material for: Child neurocognitive functioning influences the effectiveness of specific techniques in behavioral teacher training for ADHD: Moderator analyses from a randomized controlled microtrial
Source: JCPP Adv. 2021 Oct 16;1(3):e12032. doi: 10.1002/jcv2.12032 (PMC10242932; doi:10.1002/jcv2.12032)
Supplement: Supplementary file 5 — FIGURE S1 [file JCV2-1-e12032-s005.docx]

**Supporting Information Figure S1.**

Assessed for eligibility (*n* = 98)

Excluded (*n* = 8)

♦ Not meeting inclusion criteria (TTI *n* = 4, DBDRS *n* = 3)

♦ Declined to participate (*n* = 1)

Randomized (*n* = 90)

**Baseline (T0, Week 0)**

Allocated to antecedent (*n* = 30)

Completed assessment (*n* = 30)

Allocated to waitlist control (*n* = 30)

Completed assessment (*n* = 30)

Allocated to consequence (*n* = 30)

Completed assessment (*n* = 30)

**Follow-up (T1, Week 3)**

Completed assessment (*n* = 30)

Completed assessment (*n* = 28)

Teacher discontinued participation due to change of job (*n* = 1)

Child was absent due to illness (*n* = 1)

Completed assessment (*n* = 30)

**Follow-up (T2, Week 5)**

Completed assessment (*n* = 30)

Completed assessment (*n* = 29)

No assessment due to technical reasons (*n* = 1)

Completed assessment (*n* = 29)

**Analysis**

Lost to follow-up (*n* = 1)

**Figure S1.** Consolidated Standards of Reporting Trials (CONSORT) flow diagram of participants during enrollment, allocation, follow-up, and analysis.
*Note*. Analyses were intention-to-treat with *n* = 30 per condition. DBDRS = Disruptive Behavior Disorder Rating Scale; TTI = Teacher Telephone Interview.

Lost to follow-up (*n* = 0)

Lost to follow-up (*n* = 0)
