# Supplementary material for: Child neurocognitive functioning influences the effectiveness of specific techniques in behavioral teacher training for ADHD: Moderator analyses from a randomized controlled microtrial
Source: JCPP Adv. 2021 Oct 16;1(3):e12032. doi: 10.1002/jcv2.12032 (PMC10242932; doi:10.1002/jcv2.12032)
Supplement: Supplementary file 6 — FIGURE S2 [file JCV2-1-e12032-s002.docx]

**Supporting Information Figure S2.**

**Figure S2.** Task designs of the VSWMP and MFERT.

*
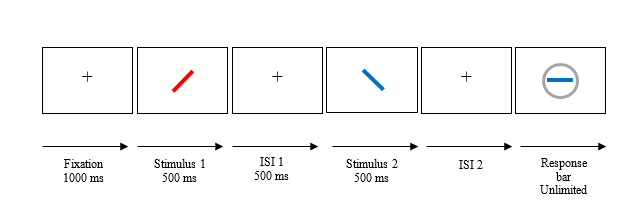
*

**Figure S2a**. Course of a trial of the Visuospatial Working Memory Precision (VSWMP) task.

**
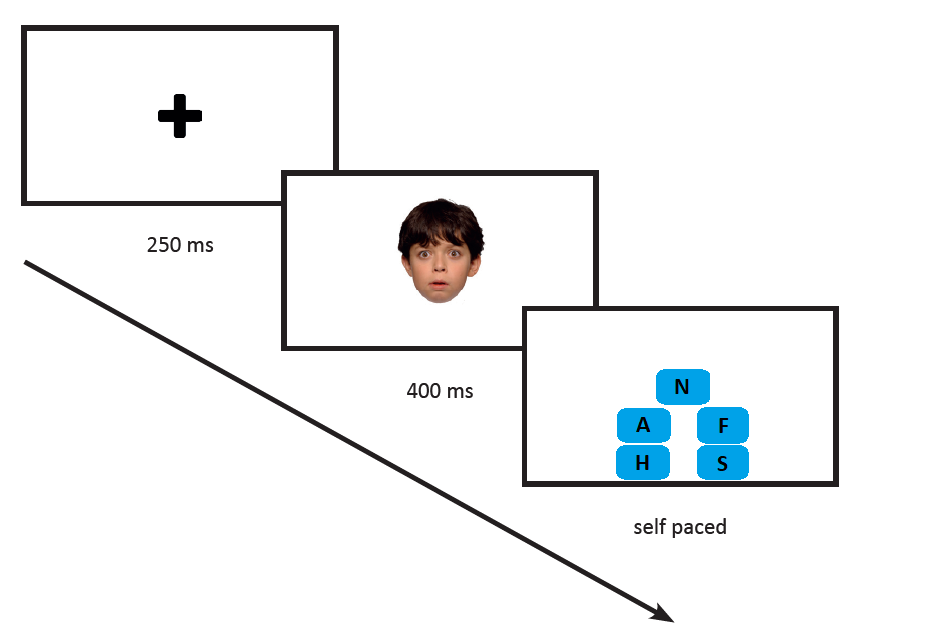
**

**Figure S2b**. Course of a trial of the Morphed Facial Emotion Recognition Task (MFERT). Abbreviations in the text boxes in the third screen represent the emotion labels anger (A), fearful (F), happy (H), sad (S) and neutral (N). Emotion labels were written out completely in the actual task (Staff et al., 2021).

*Note*. Flanker task is not depicted here because we assume this is a generally well-known task.

**References**

Staff, A. I., Luman, M., van der Oord, S., Bergwerff, C. E., van den Hoofdakker, B. J., & Oosterlaan, J. (2021). Facial emotion recognition impairment predicts social and emotional problems in children with (subthreshold) ADHD. *European child & adolescent psychiatry*, 1-13. doi:10.1007/s00787-020-01709-y
